# Development of Gastric Polyps: 15 Years of Proton Pump Inhibitor Therapy

**DOI:** 10.7759/cureus.60814

**Published:** 2024-05-21

**Authors:** Manuel Cabrera Charleston, Daniela Guadalupe Oscura Paredes, Gabriela Alfaro Mendez, Jesus Manuel Cabrera Tello

**Affiliations:** 1 Internal Medicine, Tecnologico de Monterrey, Monterrey, MEX; 2 Pathology, National Autonomous University of Mexico (UNAM), Mexico City, MEX; 3 General Surgery, National Autonomous University of Mexico (UNAM), Mexico City, MEX

**Keywords:** gi endoscopy, proton-pump inhibitors (ppi), hyperplastic polyps, gastro-esophageal reflux disease, fundic gland polyps

## Abstract

Gastroesophageal reflux disease (GERD) is a common disease affecting millions of people worldwide. Proton pump inhibitors (PPI) are the most common drugs used to treat this acid-related disorder due to their high efficacy and fewer side effects. However, long-term use of these drugs can cause histopathological changes, including cystic dilation of gastric fundic glands. The present report describes a 53-year-old man with chronic GERD and daily use of PPIs 20 mg once a day for over 15 years. This case demonstrates the association between PPI and the development of fundic gland polyps.

## Introduction

Gastroesophageal reflux disease (GERD) is a chronic condition characterized by the backflow of stomach contents into the esophagus, often accompanied by symptoms such as regurgitation and heartburn. In 2019, there were reported 783 million cases of GERD worldwide; these statistics underscore the increasing significance of this condition over the year [1]​. 

Proton pump inhibitors (PPIs) are the most common drugs for relieving GERD symptoms. The mechanism of action of PPIs involves inhibiting the gastric H+K+ATPase in parietal cells, thereby reducing acid secretion. The increasing incidence of GERD has led to a corresponding rise in the utilization of these medications [2]​. However, the use of PPIs is not without its drawbacks, as various adverse effects have been documented. Notably, prolonged exposure to PPIs has been associated with a heightened incidence of fundic gland polyps (FGPs) ​[3].

Most gastric epithelial polyps found during endoscopy are incidental and frequently exhibit hyperplastic or fundic gland histology (70%-90% of cases) [4]. FGPs may develop in association with long-term PPI use but have not been associated with an increased risk of cancer [5]. FGPs are the most common gastric polyps in patients with familial adenomatous polyposis (FAP) [6].

## Case presentation

A 53-year-old male presented to the consultation for reflux-like symptoms, including heartburn and regurgitations, for over 15 years. He complained of experiencing these symptoms all day long, but his main discomfort was at night when he presented with postprandial nocturnal acid, after dinner. To control his symptoms, he took 20 milligrams of omeprazole daily, half an hour before breakfast, for 15 years. 

Ten years ago, he had an upper GI endoscopy that revealed a type 1 hiatal hernia and a gastric polyp of unknown type. He was offered anti-reflux surgery at that time but declined.

He continued to experience reflux symptoms despite being on omeprazole for the next 10 years and thus presented to our center for evaluation. 

**Figure 1:**
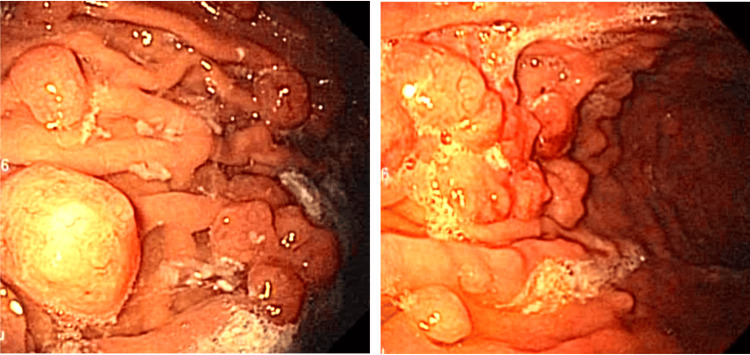
Endoscopic appearance of fundic gastric polyps.

He underwent an endoscopy where the findings were a hiatal hernia type 1 and over 30 gastric polyps. Biopsies were taken and sent for histopathological studies (Figure 1).

After the procedure, we recommended a polypectomy to rule out any malignancy or dysplasia

**Figure 2:**
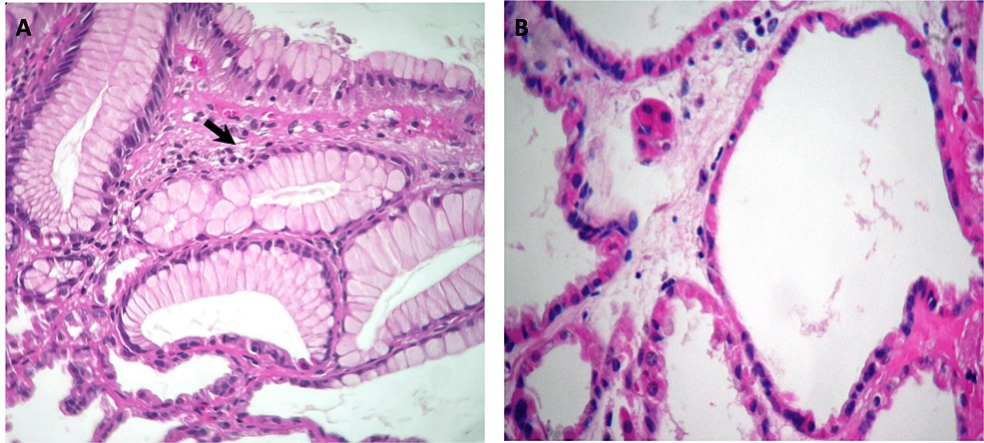
Histopathology examination of the removed polyps shows cystically dilated glands with cuboidal lining. Hematoxylin and eosin stain, magnification x40. (A) Gastric mucosa with usual columnar foveolar mucosal cells without atypia (marked by a black arrow). (B) Cystic dilation of glands lined by principal cells and some hyperplastic parietal cells, without atypia.

One week later, the endoscopic polypectomy was performed. Thirty-three polyps were removed and sent to pathology for further examination. The pathologist reported FGPs, the largest being 1.8 cm (about 0.71 inches), all of which were negative for malignancy or dysplasia (Figure 2).

The treatment for the patient was to continue with 40 mg of pantoprazole once a day and 800/10 mg of domperidone/magaldrate three times a day before any food. The patient was referred to surgery for further evaluation of an anti-reflux procedure.

## Discussion

FGPs are typically benign growths characterized by a wide base or pedicle, that protrude into the gastric cavity. They exhibit a low malignant transformation rate. PPIs inhibit gastric acid secretion, and the feedback loop that regulates gastrin release is disrupted, inducing compensatory hypergastrinemia. The reduced acidity in the stomach triggers an increase in gastrin secretion as a compensatory mechanism. This is because the normal negative feedback loop, where low gastric pH inhibits gastrin release, is no longer effective due to PPI-induced hypochlorhydria, predisposing individuals to develop FGPs ​[7]​. Chronic users of PPIs mostly develop fundic gastric polyps, a phenomenon more prevalent in Western countries characterized by lower rates of H. pylori infection and heightened PPI utilization FGPs typically manifest as small (1-7 mm), round, translucent protrusions with smooth surface [8]. They arise exclusively in the oxyntic mucosa, surrounded by an endoscopically otherwise normal mucosa. The FGPs of patients on PPIs tend to develop dilated fundic gland cysts, foveolar cell hyperplasia, and parietal cells with cytoplasmic blebs ​[9]​. PPI-associated FGPs are traditionally believed to have a low malignant potential and no ominous associations ​[10]​. Nevertheless, large polyps exceeding 1 cm in diameter warrant removal to definitively exclude malignant transformations, as these polyps rarely surpass such dimensions ​[11]​. Additionally, there were polyps with endoscopic characteristics that did not correspond in mucosa and vascularity to FGP. Some were inflammatory, while others showed alterations in their mucosa and vascularity. Given the subjectivity of endoscopy, the histopathological study provides certainty in diagnosis and classification.

## Conclusions

The presented case underscores the association between GERD, PPI therapy, and FGPs. As the incidence of GERD continues to rise globally, physicians must remain vigilant about the potential consequences of long-term PPI usage, including the emergence of gastric polyps. Although FGPs are generally benign, histopathological examination is necessary to rule out malignancy. Clinicians should inform their patients that long-term PPI use can be harmful and may not definitively alleviate their symptoms.

Recommendations include regular endoscopic evaluations for patients on long-term PPI therapy to monitor for gastric polyps and other complications. Physicians should periodically assess the necessity of continued PPI use, considering alternative treatments or step-down approaches to minimize exposure. Educating patients about the potential risks of long-term PPI use and discussing alternative GERD management strategies, such as lifestyle modifications, dietary changes, and other medications, is crucial.

This case highlights the importance of incorporating surveillance and judicious use of PPI therapy to mitigate associated adverse effects.
